# Mechanical and Assembly Units of Viral Capsids Identified via Quasi-Rigid Domain Decomposition

**DOI:** 10.1371/journal.pcbi.1003331

**Published:** 2013-11-14

**Authors:** Guido Polles, Giuliana Indelicato, Raffaello Potestio, Paolo Cermelli, Reidun Twarock, Cristian Micheletti

**Affiliations:** 1 International School for Advanced Studies (SISSA), Trieste, Italy; 2 York Centre for Complex Systems Analysis, Department of Mathematics, University of York, York, United Kingdom; 3 Max-Planck-Institut für Polymerforschung, Mainz, Germany; 4 Dipartimento di Matematica, Università di Torino, Torino, Italy; Icahn School of Medicine at Mount Sinai, United States of America

## Abstract

Key steps in a viral life-cycle, such as self-assembly of a protective protein container or in some cases also subsequent maturation events, are governed by the interplay of physico-chemical mechanisms involving various spatial and temporal scales. These salient aspects of a viral life cycle are hence well described and rationalised from a mesoscopic perspective. Accordingly, various experimental and computational efforts have been directed towards identifying the fundamental building blocks that are instrumental for the mechanical response, or constitute the assembly units, of a few specific viral shells. Motivated by these earlier studies we introduce and apply a general and efficient computational scheme for identifying the stable domains of a given viral capsid. The method is based on elastic network models and quasi-rigid domain decomposition. It is first applied to a heterogeneous set of well-characterized viruses (CCMV, MS2, STNV, STMV) for which the known mechanical or assembly domains are correctly identified. The validated method is next applied to other viral particles such as L-A, Pariacoto and polyoma viruses, whose fundamental functional domains are still unknown or debated and for which we formulate verifiable predictions. The numerical code implementing the domain decomposition strategy is made freely available.

## Author Summary

The genetic material of viruses is packaged inside capsids constituted from a few tens to thousands of proteins. The latter can organize in multimers that serve as fundamental blocks for the viral shell assembly or that control the capsid conformational transitions and response to mechanical stress. In this work, we introduce and apply a computational scheme that identifies the fundamental protein blocks from the structural fluctuations of the capsids in thermal equilibrium. These can be derived from phenomenological elastic network models with minimal computational expenditure. Accordingly, the basic functional protein units of a capsid can be obtained from the sole input of the capsid crystal structure. The method is applied to a heterogeneous set of viruses of various size and geometries. These include well-characterised instances for validation purposes, as well as debated ones for which predictions are formulated.

## Introduction

The genomic material of many viruses is encapsidated inside icosahedral protein shells with diameters in the 20–100 nm range. The number of structurally inequivalent protein units that tessellate these capsids is usually very small [1], [2]. This, in turn, is reflected in the limited repertoire of viable capsid shapes with icosahedral symmetry [3].

Understanding the organization of viral capsids at levels that are intermediate between the single protein units and the fully assembled, infectious particles is crucial to elucidate key aspects of the viral life cycle. These include the molecular basis of capsid conformational changes, such as swelling or maturation events [4], as well as the assemby/disassembly of virion particles [5]–[8]. Both these processes, in fact, are best characterised and rationalised in terms of the typically multimeric protein units [9] that behave as approximately rigid units in the capsid's conformational mechanics or act as basic assembly/disassembly units.

The identification of these units has so far been carried out for few viruses using advanced experimental or numerical techniques for probing and modelling capsids assembly/disassembly kinetics and thermodynamics, internal dynamics and response to mechanical stress [10]–[21].

These approaches have proved extremely valuable to gain insight into various mechanisms controlling the physico-chemical behaviour of few specific viruses [10]–[12], [14]–[16], [22]–[24]. For instance, nano-indentation experiments, where viral particles are subject to mechanical stress and fatigue by atomic force microscopy, have singled out the mechanical building blocks of viral capsids and elucidated the mechanisms of genome uncoating [25]. However, the systematic application of these techniques has been hindered either by the difficulty of transferring the methodologies across different virus types or by their severe experimental/computational demands.

As a step towards developing a general scheme for identifying functional and structural units in viral shells, here we introduce and apply a novel and efficient computational strategy that can single out capsid domains that, according to various criteria, are expected to be mechanically stable. The method consists of a decomposition of the capsid into quasi-rigid units based on a suitable analysis of its internal dynamics. In accord with the mesoscopic spirit of the approach, the sought internal dynamics can be efficiently obtained from elastic network approaches, in place of computationally-demanding molecular dynamics simulations.

The variational decomposition strategy is applied to several viruses covering a wide range of sizes and capsid classes, from T = 1 to pT = 7. For validation purposes, the set includes several well-characterised instances: the cowpea chlorotic mottle virus (CCMV), the MS2 virus, the satellite tobacco necrosis virus (SNTV) and satellite tobacco mosaic virus (STMV). The units obtained from the decomposition are in excellent agreement with known basic blocks of the assembly/disassembly process or of the structural transitions.

These successful comparisons give confidence in the viability of the strategy for identifying putative functional units of viral capsids. This suggests that the method could be profitably used for interpreting viral assembly, disassembly and genome uncoating experiments or as a predictive tool. Towards this latter goal, we conclude the present study by formulating predictions for a number of viruses whose capsid structure is available but whose functional units are still unknown, or debated. This prediction set includes the L-A (pT = 2), Pariacoto (T = 3) and polyoma viruses (pT = 7).

The decomposition algorithm, which is formulated in a general and hence transferable way, is made freely available for academic use at the link: http://people.sissa.it/~michelet/vircapdomains.

## Results/Discussion

The main objective of this study is to investigate whether, and if so how, a suitable analysis of quasi-rigid domains of fully-assembled viral shells can identify the functional units of a capsid. With this term we refer to those protein domains that are either: (i) the basic, undeformable building blocks (capsomeres) that can be used to describe the structural transitions of a capsid or (ii) its fundamental assembly/disassembly blocks. Although, for brevity, these two unit types are collectively referred to as “functional units”, their clear distinction must be borne in mind [9], [15], [16], [26].

The quasi-rigid decomposition approach is motivated by the observation that the large-scale internal dynamics of proteins, or protein assemblies, is often well-described by the relative rigid-like motion (rotations and translations) of a limited number of subdomains [27]–[34].

Based on this observation and building on the successful multiscale or coarse-grained simulations of viral shells modeled as assemblies of rigid tiles [15], [35]–[37], one can expect that protein capsids can be viably decomposed into quasi-rigid domains. Because of their intrinsic mechanical stability, these quasi-rigid protein units are expected to be functionally relevant.

We accordingly performed quasi-rigid domain decompositions of several viral capsids for which the atomic structural data is publicly available [38], namely CCMV, MS2, STNV, STMV, as well as L-A, Pariacoto and polyoma virus. The whole set covers various capsid geometries, namely T = 1, pT = 2, T = 3, and pT = 7, and spans a wide range of sizes, from the 60 proteins of STMV (with a total of 8820 amino acids) to the 360 ones of polyoma virus (totaling 129060 amino acids).

**Figure 1:**
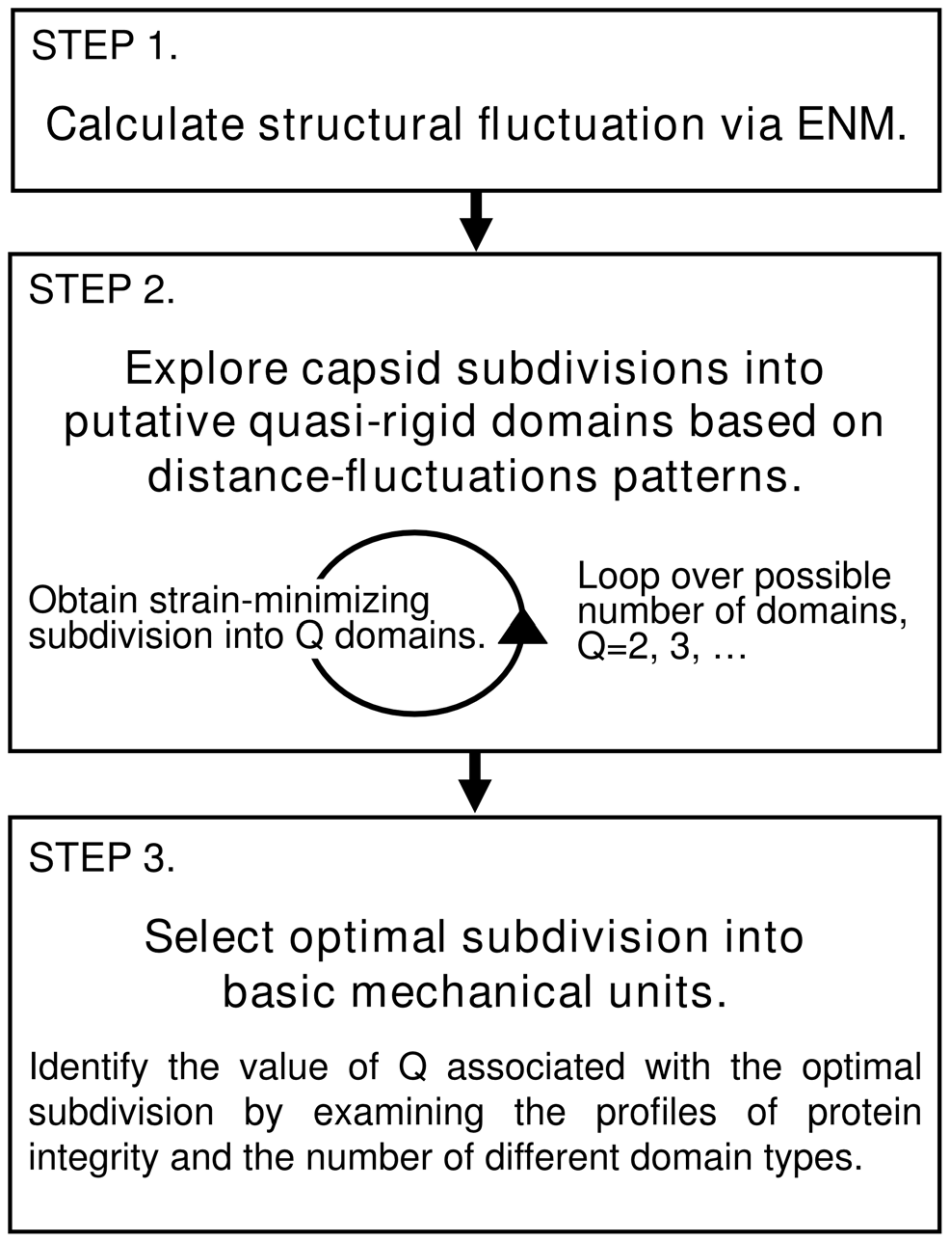
Flowchart describing the three main steps of the algorithm.

The decomposition algorithm is detailed in the Methods section and is briefly outlined here in order to convey the salient methodological steps, with their advantages and limitations. Our analysis, which follows the approach of [33], [34], [39], involves the three main steps summarised in the flow chart of Fig. 1 and briefly discussed hereafter.

1. **Calculation of structural fluctuations via ENM.** The first step consists of characterizing a capsid's internal dynamics using an elastic network model (ENM). As detailed in the Methods section, these models are based on a quadratic approximation of the free energy landscape which, by construction, has its minimum in correspondence of the reference crystal structure of the molecule [28], [40]–[45]. The viability of these models to capture the large-scale, low-energy structural fluctuations of equilibrated proteins and protein complexes has been demonstrated in several contexts by successful comparison with experimental data [22], [46], [47] and atomistic molecular dynamics simulations. The latter include instances where ENMs were applied to viral capsids [7], [8]. In fact, because of the major challenges posed by studying even small viral particles using atomistic molecular dynamics simulations [15], [16], several studies have previously relied on the use of ENMs to characterise the internal dynamics of several capsids [5]–[8], [48]. It is important to recall that in all cases, ENMs were applied to the empty protein shells. Notice that the latter may not necessarily be stable on their own *in vivo* [22], [47] (or *in silico* when realistic force fields are used [15], [16], [49]–[51]). Yet, their consideration in ENM contexts appears justifiable because the stability of the empty capsid is guaranteed by construction and hence can effectively make up for the stabilizing interactions of coat-proteins and packaged nucleic acids (typically non-resolved in available crystal structures).
2. **Exploration of capsid subdivisions into putative quasi-rigid domains.** Second, the ENM-based structural fluctuations are analysed to identify the putative quasi-rigid domains. Specifically, the capsid is subdivided into non-overlapping groups of amino acids whose internal pairwise distances have negligible fluctuations compared to the overall capsid motion. Because the optimal, “innate” number of quasi-rigid units is not known *a priori*, we consider all possible capsid subdivisions into 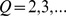 domains. For each value of 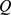, the possible amino acid partitions into 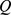 distinct groups are explored and the one which minimizes the intra-group geometric strain is identified. We note that the exploration of the combinatorial space of the possible amino acid grouping is done stochastically in a completely unsupervised manner. In particular, the groups are not constrained *a priori* to be uninterrupted in sequence or compact in space, nor to coincide with entire proteins.
  As detailed in the Methods section, the quasi-rigid character of the returned subdivision can be assessed by considering the relative weight of the two independent contributions to the overall capsid motion coming from: (i) the rigid-like relative movement of the putative quasi-rigid domains, which consists of relative rotations and translations, and (ii) the internal structural fluctuations of the groups. Clearly, for genuine quasi-rigid decompositions the rigid-like movements of the domains ought to capture a substantial fraction of the overall capsid motion.
3. **Selection of an optimal subdivision into basic mechanical units.** Finally, several order parameters are examined to identify the most plausible subdivision of the capsid into mechanically-stable units. In principle, the optimal subdivision could be identified by examining how the internal strain of the putative quasi-rigid domains decreases with 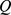. However, because such decrease is usually gradual, it is more appropriate to identify the natural quasi-rigid partition by considering a few general properties that can more sensitively discriminate between functionally viable and non-viable subdivisions.
  Arguably, a minimal set of *desiderata* for the optimal, basic mechanical units is that: (i) they should preserve the structural integrity of proteins (or protein domains), (ii) it should be possible to group them into only few structurally inequivalent types, (iii) they cannot be further partitioned into smaller units that meet the two previous criteria. Accordingly, among the strain-minimizing subdivisions for varying number of domains 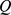 we shall pick the one which best satisfies criteria (i) and (ii), and has the smallest units, i.e. the largest 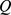.

In the next section we present the application and validation of this strategy to four viruses, namely CCMV, MS2, STNV and STMV, whose functional units have been established in previous studies [10], [52]–[58].

### Validation cases

#### CCMV

We start by considering the cowpea chlorotic mottle virus, which is well suited for validation purposes because it has been extensively studied both experimentally [26], [46], [53], [54], [59] and computationally [5], [6], [14], [60], [61].

**Figure 2:**
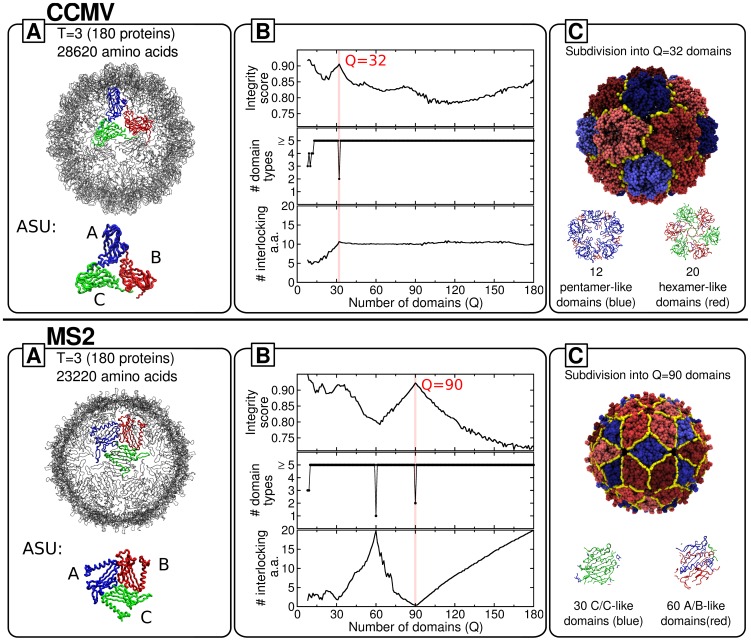
Decomposition into basic mechanical units of CCMV and MS2 viral capsids. Each left (A) box shows the capsid structure and its asymmetric structural unit (with distinct quasi-equivalent proteins highlighted in different colors). The middle (B) box shows the order parameters used to identify and characterize the optimal quasi-rigid subdivision. The latter is marked by the red dropline. The corresponding partition into basic mechanical units is represented in the rightmost (C) box. The yellow line marks the boundary between the mechanical units which, for both capsids, come in two different types and are colored in shades of blue and red, respectively. The relationship between the mechanical units and the structurally-inequivalent proteins is illustrated at the bottom of box C.

CCMV is an icosahedral RNA plant virus whose capsid is constituted of 180 chemically identical protein subunits assembled in the shape of a truncated icosahedron with T = 3 geometry. The protein units adopt three different, quasi-equivalent conformations, conventionally denoted as A, B and C [26], [53]. As shown in Fig. 2, the A proteins are organised in groups of five around the five-fold symmetry axes, whereas the B and C proteins cluster alternately in groups of six around the three-fold axes. The pentamers and hexamers are stabilised by the interactions between the N-terminal arms of the constituent subunits. These intra-capsomere interactions are complemented by inter-capsomere ones resulting from the mutual interlocking of the C-terminal arms and the 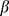-barrel of neighbouring protein pairs in different capsomeres [26].

According to various experiments these dimers correspond to the capsid assembly blocks for the virion [26], [54]. In the fully-assembled shell the dimeric units involve A/B and C/C pairs in a 2∶1 ratio. It should be noted that for A/B and C/C dimers the relative positioning of the subunits (specifically their canting angle) is different. Indeed, the subunit interlocking provides a flexible hinge that, in response to suitable environmental conditions, allows the virion to expand [59]. This fact aptly clarifies that the assembly/disassembly units are not necessarily expected to have sufficient rigidity to become the fundamental mechanically-stable units in the assembled capsid [62].

Indeed, for CCMV various studies consensually indicate that these mechanical units correspond to the pentameric and hexameric capsomeres [26], [46], [53], [54]. This conclusion can be drawn by considering the details of both the expansion process and the capsid's response to nano-indentation. In fact, during the expansion produced by the hinge-motion of the dimers, the pentameric and hexameric capsomeres rotate about their axis maintaining an internal quasi-rigid character [61].

In accord with this result, recent coarse-grained simulations of CCMV nanoindentation have demonstrated that mechanical failure occurs along the seams that bridge hexamers and pentamers, which remain largely undeformed by the application of mechanical stress [12], [14].

The above-mentioned phenomenology provides a clear context for benchmarking the proposed strategy for identifying mechanical units in viral capsids. Specifically, for CCMV it ought to return hexamers and pentamers, and not the dimers, as the primary quasi-rigid blocks.

We started by characterising the internal dynamics of CCMV by computing its collective low-energy modes of structural fluctuations and used the data to partition the capsid into a number of putative quasi-rigid units, 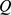, ranging from 2 up to 180 (the latter corresponding to the number of capsid proteins). The value of 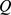 corresponding to the most plausible subdivision into functional units was found by assessing their compliance with the aforementioned *desiderata*: the preservation of protein structural integrity and the small number of structurally-inequivalent domain types.

To this purpose we computed and analysed the order parameters shown in Fig. 2. We start by discussing box B, which reports the profile of the protein integrity order parameter as a function of the number of imposed quasi-rigid domains, 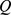. The integrity parameter is evaluated by first computing for each protein the largest percentage of its amino acids that are assigned to the same quasi-rigid block and next averaging this fraction over all proteins. Accordingly, an integrity score of 0.8 implies that, on average, 80% of the amino acids of any protein are in the same quasi-rigid block. We point out that measuring the integrity score at the level of entire proteins is appropriate for CCMV (and the other considered viruses too) because of the structural compactness of its constituent proteins. When the latter comprise two or more structural domains, the score can be straightforwardly generalised to capture the integrity of these subdomains. One such example is given by the subdivision of the Hepatitis E virus-like particle discussed in Fig. S1.

It is seen from Fig. 2 that there exists only one prominent peak of protein integrity (90%) corresponding to a subdivision into 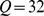 domains. The genuine quasi-rigid character of the domains is confirmed by the fact that about 85% of the capsid's mean square fluctuation results from the relative rigid-like motion of the domains, see Fig. S2. Furthermore, throughout the considered range of subdivisions, 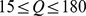, the strain-minimizing partition into 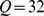 domains is the only one yielding a limited number of inequivalent domains and can be readily singled out by visual inspection. Specifically, it involves only two distinct domain types, while a minimum of 5 to a maximum of 23 different types is found for all other values of 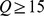. Lower values of 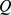, which correspond to subdivisions into very few macrodomains, are more obviously associated to both high integrity scores and few different domain types, see Fig. S3.

The combined inspection of the integrity score and the domain types therefore provides a clearcut and non-ambiguous indication of the “innate” character of the CCMV capsid subdivision into 32 quasi-rigid domains which in turn can be grouped into only two structurally inequivalent types. The corresponding subdivision is shown in box C of Fig. 2, with the two domain types colored in shades of blue and red, respectively. The inspection of the subdivision shows that one domain type corresponds to pentameric units and the other to hexameric ones. There are 12 and 20 domains of each type, respectively. By considering the detailed structural representation of the two domain types, shown at the bottom of box C in Fig. 2, it is readily seen that they are, practically, an exact match of the hexameric and pentameric capsomeres described before, the only difference being that the interlocked C-terminus is assigned to the “host” dimeric subunit and not to the parent one. The swapping of C-termini across the hexameric and pentameric units yields an integrity score smaller than 100%.

A further relevant parameter to consider for assessing the functional role of the subdivision is the degree of domain interlocking. The corresponding profile is shown in the bottom graph of box B in Fig. 2 and portrays the average number of a protein's terminal amino acids assigned to a quasi-rigid domain which is not the one containing the protein core.

This parameter is monitored because several viruses, including CCMV, are assembled from protein dimers stabilised by the mutual interlocking of their termini which reach inside the partner protein core. The incidence of such interlockings *across* different quasi-rigid domains provides valuable clues regarding the relationship between the mechanically stable domains and capsid assembly/disassembly. In particular, the absence of cross-domain interlocking ought to be a good indicator that the mechanical domains are viable assembly/disassembly units too. The opposite should hold in case a significant amount of cross-domain interlocking is observed. It should, nevertheless, be borne in mind that cross-domain interlocking can arise after the assembly process.

For the case of CCMV, we observe that the degree of inter-domain interlocking for 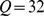 is non-negligible and, indeed, it reflects the above mentioned dimeric swapping of the C-termini between protein subunits. From the previous considerations, this fact indicates that the quasi-rigid hexamers and pentamers do not have the correct level of internal structural independence to be viable candidates for assembly or disassembly blocks. This conclusion is indeed correct given the known role of dimers with linked domains as assembly units.

In conclusion, the emerging quasi-rigid domain subdivision matches correctly the units identified by previous experimental and numerical studies.

#### Bacteriophage MS2

We next consider the MS2 virus, which is constituted by 180 chemically-identical coat proteins with a total of 23220 amino acids [63], [64]. As for CCMV, the protein units come in three structurally-inequivalent types (conformers), labelled A, B and C in box A of Fig. 2, which form interlocked A/B and C/C dimers and are assembled in a T = 3 capsid geometry. However, the arrangement of these units is different: the asymmetric A/B dimer occurs in two groups of 5 around the 6 five-fold axes, and the symmetric C/C dimers are positioned on both ends of the 15 two-fold axes.

The results of the MS2 quasi-rigid domain subdivisions are illustrated in the upper panel of Fig. 2. The protein integrity profile shows one prominent peak corresponding to the subdivision into 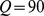 quasi-rigid blocks, whose relative rigid-like motions suffice to capture about 95% of the capsid's mean square fluctuations, see Fig. S2. These quasi-rigid units come in only two inequivalent types, as illustrated in box B. Detailed inspection of the subdivision reveals that these two types occur precisely in a 2∶1 ratio and correspond to the C/C and A/B dimers, which are colored in shades of blue and red, respectively, in box C. As before, this match of the mechanical domains and structural dimers must be understood with the proviso that protein integrity cannot be fully respected. In fact, amino acids at the boundary of quasi-rigid dimer domains are not necessarily assigned to their sequence-wise nominal dimer. As a result, although the whole A/B and C/C dimers would comprise exactly the same number of amino acids, the two types of quasi-rigid units are structurally diverse enough to be distinguishable by size, see box C in Fig. 2.

It is worth recalling that the MS2 capsid is in the same T = 3 class as CCMV. Hence their very different number and types of fundamental quasi-rigid units point to the important role played by specific capsid proteins in shaping the properties and behaviour of viral capsids that are not large enough to be dealt with by continuum approaches [9]. One further major difference between the MS2 and CCMV optimal subdivisions is that the 90 units have a practically negligible degree of interlocking. Indeed, the interlocking profile has a minimum for 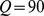. This indicates that the small quasi-rigid units are structurally self-contained dimers. They are therefore viable candidates for being not only the fundamental mechanical blocks of the fully-assembled capsid but can be expected to be structurally-stable even in isolation and hence are also good candidates for being the assembly or disassembly units of the capsid. Indeed, this has been confirmed by isotope pulse-chase experiments [56]. In these experiments, protein subunits of dimers in complex with RNA are labelled differently from those in RNA-free dimers (via different isotopes) and both species are mixed. The fact that no dimers with differently labelled subunits are detected in solution or as part of any of the assembly intermediates suggests that the dimers do not fall apart into individual subunits and that hence the dimer is indeed the unit of assembly.

We emphasize that this *a priori* conclusion has necessarily a tentative character. In fact, because the method is based on the properties of fully-assembled protein shells, it cannot account for the interaction of coat proteins and genomic material during the assembly process. Such interaction can be crucial to aid the fast and correct assembly *in vivo* [36], [37], [55], [56], [65]–[67]. However, building on the fact that spontaneous *in vitro* assembly does occur in the absence of the genome, it appears plausible to consider non-interlocked quasi-rigid units as putative assembly units.

These considerations are fully supported by the successful comparison with experimental data for MS2. In fact, it has been established that the capsid is assembled from the A/B and C/C dimeric units [56], and the assembly pathways have been characterized in detail both experimentally and theoretically [10], [11]. In addition, the key role of the dimeric protein-protein interactions for capsid stability has been indicated by thermal and pressure denaturation experiments [68].

In summary, the MS2 findings reinforce the CCMV indications that the innate functional units identified with the quasi-rigid domain decomposition correspond to those established experimentally.

#### STNV

The satellite tobacco necrosis virus has been one of the first to be determined at high resolution [69], [70]. With a diameter of only 17 nm, this T = 1 RNA plant virus is one of the smallest known. The capsid is composed of 60 chemically and structurally identical coat proteins. Each of these consists of 195 amino acids and their N-terminal arms are positively charged [52], [58], a common feature in many plant viruses. In the fully-assembled, genome-loaded capsids (which are extremely stable [15], [58]) the N-termini interact with RNA loops, achieving charge neutrality. This interaction has been argued to favour an extended and ordered conformation of the N-termini, which in turn aids the formation of trimeric capsomere units [52], [57], [71].

**Figure 3:**
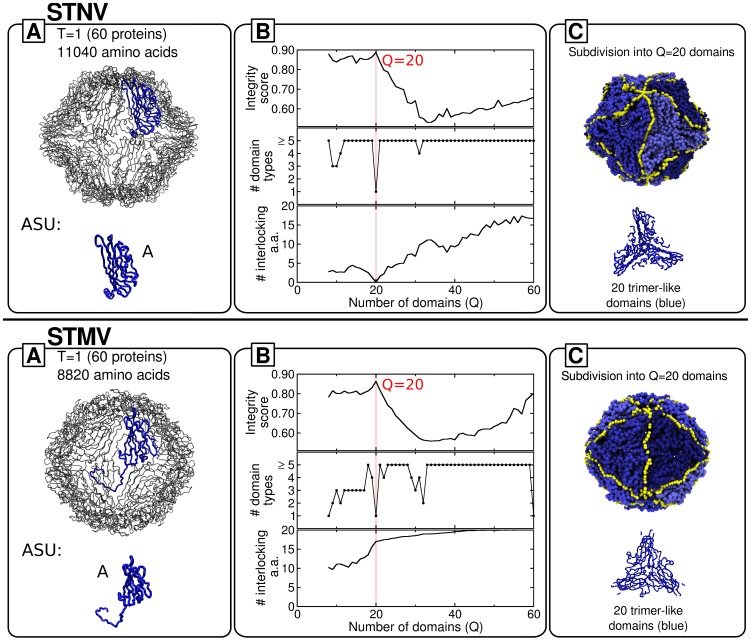
Decomposition into basic mechanical units of the STNV and STMV capsids. Boxes A, B and C, show respectively the capsid structural organization, the profiles of various order parameters and the optimal decomposition into basic mechanical units. Colors and capsid representations follow the same scheme as in Fig. 2.

The quasi-rigid domain decomposition, whose results are reported in Fig. 3, returns an optimal subdivision for 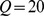 mechanical domains. Their relative rigid motion accounts for more than 60% of the capsid's structural fluctuations, see Fig. S2. These domains correspond to trimeric units that are monodisperse in size and do not have interlocked termini. This outcome is consistent with the assembly mechanism discussed above, which involves 20 trimers as basic assembly units [52].

A noteworthy implication is that the fundamental units of the assembly process, in which the RNA is known to play a major role, can be correctly identified through the quasi-rigid domain decomposition of the empty capsid. In this regard, it must be borne in mind that elastic network models guarantee by construction the stability of the model capsid for structural fluctuations of the crystal structure. Therefore, as remarked earlier, ENM approaches can make up for the missing stabilizing interaction of capsid proteins and the packaged nucleic acid. At the same time, the finding indicates that the mechanical stability of the individual (non interlocked) assembly units is still discernible in the internal dynamics of the fully-assembled capsid.

This remarkable property shows *a posteriori* that even in cases where protein-nucleic acids interplay is important, the quasi-rigid domain analysis of the pure protein shell can still give valuable clues about the assembly process.

#### STMV

It is interesting to compare the above analysis with the one for another plant virus, the satellite tobacco mosaic virus (STMV), which presents several similarities with the STNV [15], [58] including the T = 1 arrangement of the 60 identical coat proteins (with a total of 8820 amino acids) [72].

Because of its relatively small size, STMV represents an ideal and natural reference for numerical investigations [15], [16]. To the present day, it remains the only virus for which all-atom molecular dynamics simulations have been performed on the fully-assembled capsid, both in the presence and in the absence of the genome [16].

This study as well as coarse-grained simulations [15] provide considerable insight into the internal dynamics of the capsid, its structural stability and resistance to nanoindentation. The consensus indication of these investigations is that the basic mechanical units are trimers of coat proteins.

While this represents a further point of contact with STNV, it should be noted that the similarity of their assembly processes is still disputed. In fact, it is not yet understood whether assembly proceeds as a condensation of a protein-RNA complex [73] or if the collapse of the RNA into a globular state precedes and favours the formation of trimeric and pentameric units [16].

The results of the quasi-rigid domain decomposition of STMV are provided in the bottom panel of Fig. 3. The profiles shown in box B provide a clear indication that the basic rigid units correspond to monodispersed (identical) trimers; this partitioning of the capsid suffices to capture as much as 85% of the capsid's structural fluctuations, see Fig. S2. This result is fully consistent with the previously mentioned computational studies of STMV's structural stability, and also parallels the results of the related STNV case.

However, at variance with STNV, the analysis of the interlocking profiles shows that, at low values of 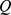, the trimers present a significant degree of interlocking originating from the interdigitating N-terminal arms of dimers that straddle domain boundaries. This difference from STNV is not surprising, given the lack of amino-acid homology or immunological cross-reactivity between STMV and STNV [74]. As previously discussed for CCMV and unlike STNV, the significant interlocking prevents from concluding that the trimers are plausible building blocks for the assembly of STMV.

As a matter of fact, McPherson *et al.* [74] suggest that the building blocks may be dimers that contact the genomic RNA at the particle 2-fold axes. This open issue could possibly be settled by establishing whether termini interlocking occurs before or after assembly. This information, which is at the heart of the ongoing debate on the STMV assembly process, is clearly beyond reach of the present approach which is based only on the fully assembled capsid.

### Predictions

We now turn to discuss three viruses for which the basic, mechanically stable functional units are not conclusively known. The following viruses are considered, chosen in order of increasing complexity of the capsid type (T-numbers): the L-A (pT = 2), Pariacoto (T = 3) and polyoma (pT = 7) viruses. We recall that the pT = 2 and pT = 7 cases refer to non-standard Caspar-Klug geometries.

#### L-A virus

**Figure 4:**
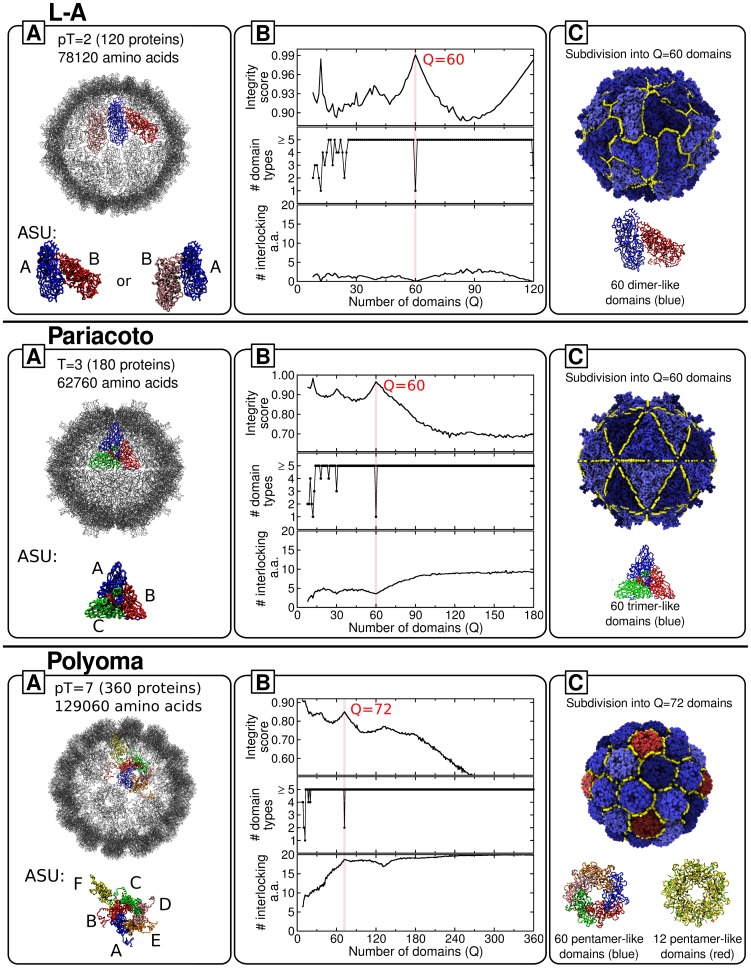
Decomposition into basic mechanical units of L-A, Pariacoto and polyoma viral capsids. Panels are organised as in Fig. 2. Boxes A, B and C, show respectively, the capsid structural organization, the profiles of various order parameters and the optimal decomposition into basic mechanical units. Colors and capsid representations follow the same scheme as in Fig. 2.

The L-A virus is a double-stranded RNA (dsRNA) yeast virus whose capsid is composed of 120 chemically-identical coat proteins with a total of 78120 amino acids. The proteins are assigned to two types, A and B, based on their inequivalent positions, see box A in Fig. 4. Similar to several other dsRNA viruses, the A/B dimers are arranged in a T = 1 icosahedral capsid. This virus is classified as pT = 2 to account for the fact that dimers occupy the positions of monomers in a T = 1 structure [75], [76]. Stable empty capsids are observed *in vitro* and it has been suggested that A/B dimers are the basic assembly building blocks [75]. By inspection one readily recognizes that the A/B asymmetric unit tiling the capsid can be defined in two inequivalent asymmetric ways (see Fig. 4). Because the two alternative pairings have a comparable buried surface area, it is not clear *a priori* which dimer type could be the basic assembly block. As we discuss hereafter, the quasi-rigid domain analysis can provide valuable insights into this open problem.

From the analysis of the plots in box B of in Fig. 4 it emerges very clearly that the optimal subdivision is attained for 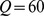 identical quasi-rigid domains. Their relative rigid motion captures about 80% of the capsid's mean square fluctuations, see Fig. S2. Because of the high integrity score of this subdivision and the bipartite A/B capsid tiling, it follows that these basic mechanically-stable units necessarily correspond to A/B dimers which, furthermore, are negligibly interlocked.

This result is therefore fully consistent with the experimental indication of A/B dimers being the basic assembly units.

The notable point is that the quasi-rigid domain analysis discriminates very clearly between the two inequivalent asymmetric A/B dimers shown in box A, arguably because of their different networks of intra- and inter-dimer interactions. In fact, upon repeating the quasi-rigid domain partitioning into 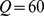 domains, one invariably observes that the strain-minimizing subdivision is the one shown in box C of Fig. 4. Given the robustness of this subdivision we predict that the A/B dimer shown in box C is the basic assembly unit of the L-A virus.

#### Pariacoto virus

The Pariacoto insect virus belongs to the nodaviridae family and has a T = 3 capsid [77], [78] constituted by 180 chemically identical coat proteins occupying three quasi-equivalent positions. As shown in Fig. 4 the A units cluster around the five-fold axes while the B and C units are found at the three-fold axes. The capsid consists of 62760 amino acids in total.

While the C-terminal arm of each A protein is located in a channel formed by the A, B, C monomers at the quasi-3-fold axes, the N-terminal arms of the A proteins are involved in an extensive interaction with the encapsidated single-stranded RNA [77].

The inspection of the profiles in box B of Fig. 4 indicates that the optimal subdivision into mechanically-stable units is obtained for 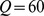, whose rigid-like motion accounts for about 90% of the capsid's structural fluctuations, see Fig. S2. This partition corresponds to monodispersed, identical trimers, see box C. The other prominent peak for the much smaller number of 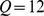 subdivisions corresponds to multiples (pentamers) of these trimeric units. The trimeric units correspond precisely to the A, B, C complexes and their minimal degree of interlocking is suggestive of their role as basic assembly units for the Pariacoto virus capsid.

The identification of a trimer of proteins as the first stage of assembly is also consistent with the theoretical work by Reddy [79], which is based on calculations of the buried surface area of the coat proteins.

#### Polyoma virus

We conclude the analysis with the discussion of the murine polyoma virus. This non-enveloped DNA virus has an icosahedral capsid with a pT = 7 (non Caspar-Klug) geometry [80]. The shell consists of 360 copies of the main coat protein (VP1) with a total of 129060 amino acids, the largest capsid considered here. The asymmetric structural unit involves six identical coat proteins which are organised into pentameric clusters with structurally inequivalent bonding environments [81], see Fig. 4.

The peak structure of the integrity score profile indicates that the optimal subdivision involves 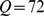 rigid domains. Their rigid-like motion accounts for about 90% of the capsid's structural fluctuations, see Fig. S2. As illustrated in box B of Fig. 4, these correspond to pentamers. More precisely, two inequivalent types of pentamers are recognized by our approach. The pentameric units shown in box C are therefore expected to be the stable mechanical units for the capsid (though not the assembly ones because of the significant amount of interlocking).

This conclusion is reinforced by the analysis of the suboptimal subdivision into 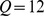 domains. These larger domains correspond to five-fold symmetric units made of a central pentamer surrounded by five further pentamers and hence give additional support to the capsid's flexibility at pentamer-pentamer boundaries. This prediction could be verified by e.g. using molecular dynamics simulations to analyse the response of the capsid to nano-indentation.

### Summary and conclusions

Identifying the fundamental, and typically multimeric, protein units that control the mechanical response of viral capsids or its assembly and disassembly is important both for rationalizing and for modeling key steps of viral life cycles [15].

Here we introduced and applied a novel computational strategy that, to our knowledge, represents the first attempt to develop a general and efficient method for identifying the basic, mechanically stable protein units starting from the sole input of the fully-assembled protein capsid. The method relies on the characterization of the internal dynamics of the capsid by means of elastic network models and uses it to optimally decompose the protein shell into blocks that have the characteristics expected for genuine capsid functional units, such as mechanical stability (quasi-rigidity), structural integrity of the constitutive proteins, or small numbers of inequivalent block types etc.

The viability of the scheme was first assessed and validated by considering a set of four viruses (CCMV, MS2, STNV, STMV) for which the fundamental functional units are known. In all cases, the results of the optimal decomposition scheme were fully consistent with available experimental or numerical results for the known mechanical and/or assembly protein units. We next turned to a further set of three viruses, namely polyoma, Pariacoto and L-A virus, whose functional units are debated or not known, and for which we formulate verifiable predictions.

The positive validation of the method and its affordable computational cost (the first hundred ENM modes of the internal dynamics of capsids of about 60000 amino acids can be obtained in 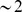 hours on a single Intel Xeon 2.40 GHz processor) demonstrate that simple structure-based strategies can provide considerable information on the basic functional units. In particular, they not only aid the understanding of various viral processes but can also guide the development of their multiscale modelling.

We envisage two natural extensions of this first study. On the one hand it would be important to explore the possibility to include, even approximately, the interaction of the coat proteins with the packaged genome. This would be an apt complement of previous studies which considered the viability of ENM characterizations of empty capsid shells as proxies for the genome-loaded virion particles. On the other hand, it would be most interesting to extend considerations systematically to larger and more complex capsid geometries in order to understand how the functional units change as one goes from small- or medium-sized capsids (where the discrete protein nature of the capsid is visible) to larger structures that are well approximated by continuum theory [9].

## Methods

### Calculation of the structural fluctuations via ENM

Proteins and protein assemblies in thermal equilibrium can sustain structural fluctuations of appreciable amplitude. A large body of experimental and numerical evidence has indicated that the principal fluctuation modes, those of lowest energy, have a collective character. This means that the structural deformations associated to these modes entail the concerted displacements of groups of several amino-acids.

As was first shown by Tirion [41], the collective character of the modes justifies the use of simplified, coarse-grained models (rather than atomistically-accurate ones) for calculating the principal modes of fluctuation of a protein around its reference, native structure.

A commonly used framework for such coarse-grained calculations is provided by elastic network models. The latter rely on a quadratic approximation of the near-native protein free energy,
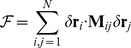
(1)where 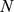 is the number of amino acids, 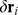 is the vector displacement from the native position of the 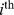 main chain (backbone) centroid (typically the 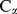 atom) and 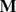 is the effective symmetric interaction matrix of linear size 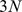.

Within the quadratic approximation of Eq. (1), the principal modes of structural fluctuations can be calculated exactly with minimal computational expenditure, and they correspond to the eigenvectors of 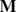 having the lowest non-zero eigenvalues.

In the following we shall indicate by 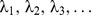, the non-zero eigenvalues ranked according to increasing magnitude (they are all positive) and with 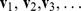 the corresponding orthonormal eigenvectors. It can be shown that 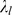 corresponds to the total mean square structural fluctuation projected on the 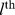 mode, 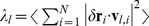, where 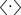 denotes the canonical equilibrium average and 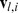 is the displacement of the 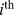 amino acid projected on the 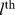 mode.

In this study, we shall resort to the beta-Gaussian network model [45] to compute the matrix 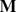 and its eigenvalues and eigenvectors. The model, which is implemented in a freely-available numerical code [45], was previously successfully validated against extensive molecular dynamics simulations of various proteins and protein complexes. At variance with most elastic network approaches it uses not one, but two interaction centers per amino acid: one for the main chain, the other for the side-chain (omitted for glycine). As customary, the centroids' interaction range was set equal to 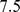 Å. Because the side-chain degrees of freedom are integrated out analytically, the linear size of the matrix 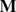 is still equal to 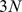, as in single-centroid schemes.

The computational burden associated with the memory storage and diagonalization of the 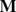 matrices for the capsids (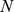 is in the 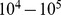 range) was limited by taking advantage of the sparse character of 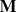 and calculating its lowest-energy eigenvectors using the shift-inverse Arnoldi method, as implemented in the Arpack routines [82]. These algorithmic techniques (which could be further aided by symmetry considerations [83]) sufficed to compute the relevant low-energy modes of all capsids, except for L-A, using less than 24 Gb of RAM and a single 2.4 GHz Intel processor. The modes calculation is the slowest computational step in the whole decomposition procedure for larger viruses (for instance, it took about 3 hours for the L-A case).

For the polyoma capsid alone which, at 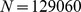, is the largest entry in our set, we found it necessary to adopt a coarser ENM description. Specifically, we used one centroid per two amino acids by retaining only one for every other 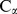. The interaction range was rescaled accordingly and set equal to 15 Å. Consistent with established results for the case of globular proteins [84], this coarse-graining procedure has no effect on the optimal quasi-rigid domain decomposition of smaller capsids. This is illustrated in Fig. S4 for the STMV capsid which, being the smallest considered here, is expected to be the most susceptible to the coarse-graining level. This validation and the considerations of [84] provide a justification for the use of the coarse-grained description for the polyoma capsid.

### Exploration of capsid subdivisions into putative quasi-rigid domains

The subdivision of viral capsids into quasi-rigid domains is based on the PiSQRD strategy introduced in refs. [33], [34]. The approach relies on the notion that for a genuine rigid-body the modulus of the distance of any two points remains constant as the body is moved in space.

**Figure 5:**
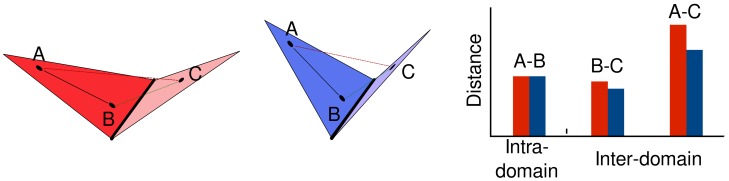
Schematic illustration of the relative motion of two rigid domains (triangles) joined by a hinge. Two possible configurations are colored in red and blue. The graph on the right illustrates the distances of various pairs of points in the two configurations. The A/B intra-domain distance does not vary while A/C and B/C inter-domain distances do.

Accordingly, one can quantify the viability of a tentative capsid subdivision into 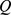 putative quasi-rigid domains by comparing amino acids' pairwise distance fluctuations within each domain with those across domains. For good subdivisions, the former should be much smaller than the latter, see sketch in Fig. 5.

To turn this observation into a quantitative scheme amenable to numerical implementation, we consider the geometric strain, 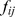, for a given pair of amino acids, 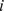 and 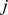. Using the same notation introduced after equation (1) for the principal modes of structural fluctuations, 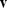, and their associated amplitudes 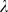,
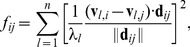
(2)where 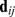 is the reference, native distance vector of the 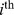 and 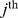 amino acid, and 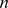 is the number of retained principal modes.


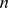
 is chosen by retaining all the modes with energy lower than the fifth non-zero mode of a single coat protein, thus ensuring a sufficient level of detail while minimizing the computational effort and discarding the mostly irrelevant high-frequency details.

Accordingly, the internal strain of the 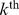 domain 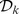 is defined as
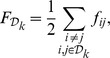
where the sum runs over all the pairs belonging to that domain, and the overall strain is therefore
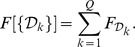
Based on previous considerations, the desired subdivision is the amino acid partitioning into 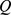 groups that minimizes the overall strain 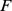. Notice that the minimization of 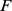 needs to be performed separately for all possible values of 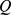, that is from 2 up to the number of protein units forming the capsid (or even larger values in case the mechanical domains involve protein structural subunits). In fact, the “correct” optimal number of quasi-rigid domains is not known *a priori* and needs to be found based on physical considerations, see the next subsection.

For each explored value of 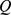, the minimization of 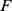 over the amino acids' assignments is performed by a greedy algorithm starting from a random labelling. At each step of the algorithm a randomly-picked amino acid is reassigned to a randomly-chosen domain. The new assignment is accepted if it leads to a decrease of 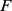 and rejected otherwise. The scheme is repeated until the algorithm is unable to further improve the solution, i.e. the count of systematically rejected moves is comparable with the total number of amino-acids.

To reduce the impact of getting trapped in local minima of 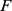 (whose landscape roughness increases with 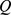) the greedy minimization scheme is iterated if the distribution of the domain strain 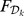, 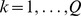 is highly heterogeneous (which could be a sign of a very asymmetric solution). Specifically, we first compute the average, 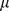, and standard deviation, 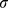, of the domains' strain and check if one or more residuals 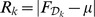 is larger than 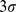. If so, then the two domains with smallest strain are joined while the one with the largest strain is split in two. This amino acid reassignment clearly preserves the total number of domains, 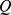. The greedy minimization of 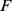 is repeated and the procedure is iterated until one of the following holds: (i) convergence to a minimum which features a sufficiently homogeneous energy distribution, or (ii) the splitting/joining move is unable to improve the solution.

It is important to note that no *a priori* information about the capsid's parsing into single proteins is used to identify the domains. Indeed, in principle mechanical domains can cut through proteins, for example when a rather loose loop tightly binds to a different block. The comparison between the mechanical and the proteins boundaries is done a posteriori, providing information on the reliability of the subdivision itself.

The 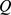-dependence of the miminized geometric strain for all considered capsids is shown in Fig. S5.

### *A posteriori* assessment of the quasi-rigid character of a subdivision

Besides calculating the geometric strain, the genuine quasi-rigid character of a given decomposition into 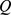 domains is more intuitively assessed by computing the fraction of overall capsid motion that can be ascribed to the relative rigid-like movements of the domains (i.e. by neglecting intra-domain fluctuations as if the domains were strictly rigid). This quantity is calculated by considering that each normalised mode, 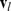, can be decomposed as a sum of two contributions: one consisting of pure rigid rotations and translations of the domains, 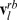, and one describing intra-domain fluctuations, 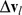, i.e. 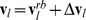. Because these two components are orthogonal [33] one has that 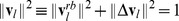. The fraction of the capsid's mean square structural fluctuations that can be ascribed to the relative rigid displacement of the domains is accordingly:
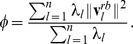
The profile of the fraction of motion captured by the domain decomposition of all considered capsids is shown in Fig. S2.

### Selection of optimal subdivision into basic mechanical units

The algorithm for the subdivision into 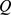 domains was applied to the viral capsid several times, varying 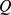 between 2 and the total number of proteins in the capsid. After establishing the quasi-rigid character of the putative subdivisions by monitoring the strain and the above-mentioned fraction of captured motion, the identification of the optimal value(s) of 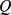 corresponding to a subdivision into viable, basic mechanical units was performed by monitoring two physical quantities: protein integrity and the number of inequivalent capsomere types. They respectively account for the compatibility of the subdivision with the natural elementary units represented by the single proteins and for the structural similarity of the tiles, which results in a low number of different tiles.

Given a subdivision into domains, an integrity parameter was defined for each protein. For a general subdivision, the amino acids of a protein can be assigned to a number of different domains. However, a good subdivision should preserve the integrity of the protein, i.e. almost the whole protein should belong to a single domain. We thus defined the integrity score for a protein as the largest fraction of its amino acids assigned to a single domain. This quantity was then averaged for all the proteins, providing a score for the capsid subdivision.

We also computed the number of similar tiles identified by our subdivision by size inspection. Specifically, we defined the size of the 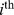 domain as the number of amino-acids belonging to the domain itself; we then assigned domains to a tile type if their size is the same within ca. 3% of the average size.

Viable subdivisions into basic mechanical units were identified by maxima in the integrity score corresponding to a small number of tile types.

### Interlocking between capsomeres

To detect possible intertwinings between quasi-rigid units (e.g. due to swapped tails or subdomains of the parent proteins) we computed the *interlocking* parameter. Specifically, we considered separately the two termini of each protein in the capsid, namely the first and last twenty amino acids, and counted the number of amino acids assigned to a rigid domain different from the dominant one (i.e. the domain to which most of the protein's amino acids belong). This calculation returned the number of interlocked amino acids for each terminus of each protein in the capsid. The numbers relative to the N and C terminals were averaged separately, and the largest of the two averages was taken as a measure of the interlocking of the quasi-rigid domains. In other words, if a quasi-rigid domain subdivision has interlocking number equal to 10, it means that on average one protein has 10 terminal residues assigned to a different domain than its core. Clearly, this also implies that the other terminus has less than 10 interlocked amino acids.

## Supporting Information

Figure S1
**Decomposition into basic mechanical units of the HEV virus-like particle.** As is shown in box A, each of the 60 coat proteins features three distinct structural subdomains, named S (a coat domain which composes the envelope for the genetic material), P1 (which forms a protrusion around the three-fold axis) and P2 (which forms spikes on the two-fold axis). The optimal subdivision, corresponding to 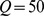 domains (coming in two distinct types) is identified by the peak in the integrity score calculated at the protein level and at subdomain level, see the black and blue curves, respectively, in box B. The fact that the peak of the subdomain integrity is much more prominent than for entire proteins indicates that the basic mechanical domains involve structural subunits from different proteins. This is clearly visible in box C which shows that one domain type corresponds to the spike (formed by the P2 subunits of two neighbouring coat proteins) while the other is a trimer involving the S and P1 subunits of three neighbouring coat proteins.(TIF)

Figure S2
**Fraction of overall capsid motion (mean square structural fluctuations) that can be ascribed to the pure rigid-like movements of the** 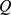 **quasi-rigid domains.** For each value of 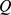 we considered the domain subdivision which minimizes the geometric strain. Panels a-h refer respectively to: CCMV, MS2, STNV, STMV, L-A virus, Pariacoto virus, polyoma virus and HEV.(TIF)

Figure S3
**Suboptimal decompositions of CCMV.** Panel A shows a close-up of the CCMV profiles for the integrity score and number of tile types for subdivisions from 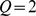 up to 30 quasi-rigid domains. Panel B illustrates non-optimal quasi-rigid decompositions of CCMV. The subdivisions correspond to partitions into very few domains as indicated by the 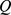 label. For each of these subdivisions the number of different tile type is large and ranges from 3 to 4. For simplicity we therefore used a different color for each domain rather than a different color for domain type as in the figures in the main text.(TIF)

Figure S4
**Structural coarse-graining and robustness of quasi-rigid domain decompositions.** Optimal subdivision of the STMV capsid into 20 quasi-rigid domains obtained by using the coarse-grained ENM where only every other 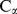 atom is retained, see Methods. The profiles of various order parameters for the subdivison are shown in box A. The resulting coarse-grained subdivision is shown in box B and is practically indistinguishable from the one given in Fig. 3 where all 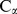 atoms were retained.(TIF)

Figure S5

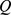
 **-dependence of the miminized geometric strain.** Panels a-h refer respectively to: CCMV, MS2, STNV, STMV, L-A virus, Pariacoto virus, polyoma virus and HEV. Notice that at the value of 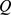 corresponding to the optimal subdivision (highlighted by the red band) there is usually a kink. The latter signals the change of the slope of the strain curves when the “innate” number of subdivisions is crossed.(TIF)
