# Supplementary figures and images for: Mechanical and Assembly Units of Viral Capsids Identified via Quasi-Rigid Domain Decomposition
Source: PLoS Comput Biol. 2013 Nov 14;9(11):e1003331. doi: 10.1371/journal.pcbi.1003331 (PMC3828136; doi:10.1371/journal.pcbi.1003331)

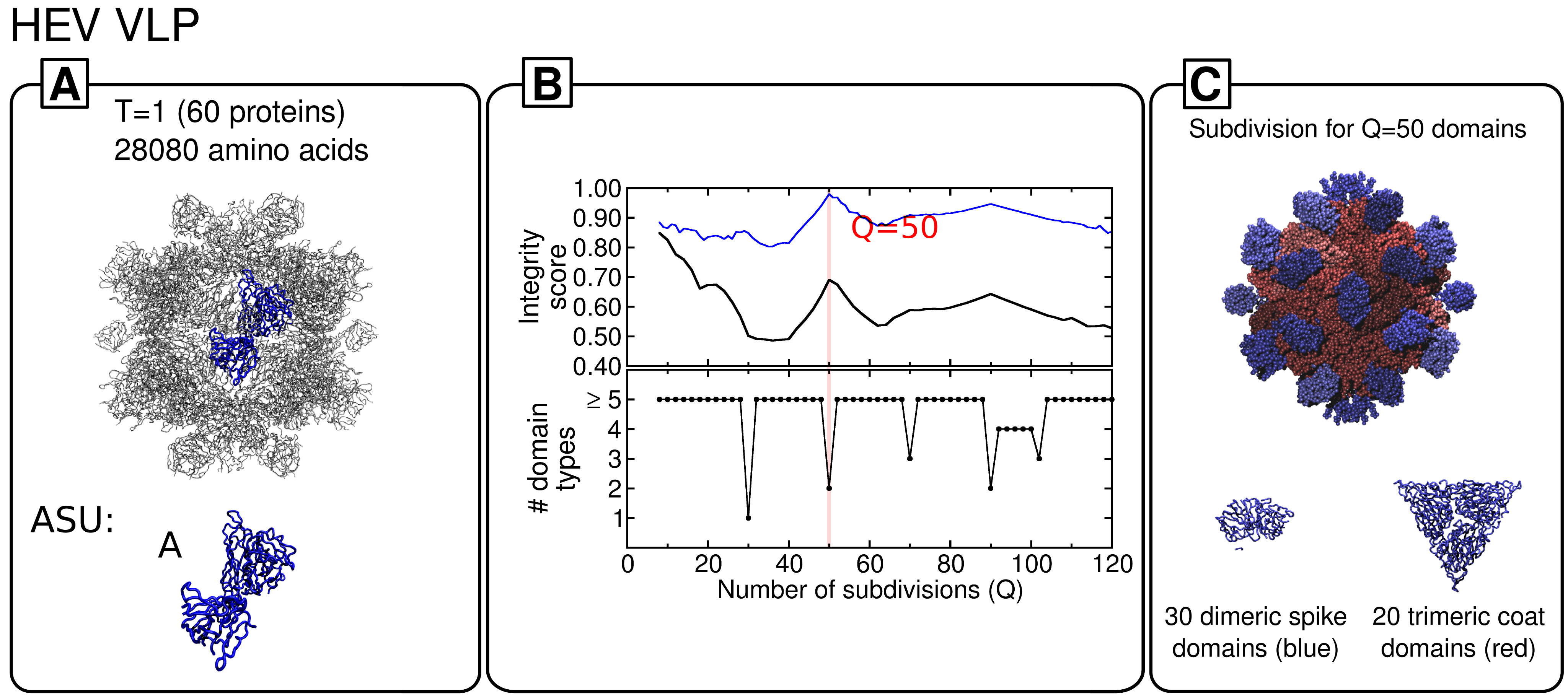

Supplement: Figure S1 — Decomposition into basic mechanical units of the HEV virus-like particle. As is shown in box A, each of the 60 coat proteins features three distinct structural subdomains, named S (a coat domain which composes the envelope for the genetic material), P1 (which forms a protrusion around the three-fold axis) and P2 (which forms spikes on the two-fold axis). The optimal subdivision, corresponding to domains (coming in two distinct types) is identified by the peak in the integrity score calculated at the protein level and at subdomain level, see the black and blue curves, respectively, in box B. The fact that the peak of the subdomain integrity is much more prominent than for entire proteins indicates that the basic mechanical domains involve structural subunits from different proteins. This is clearly visible in box C which shows that one domain type corresponds to the spike (formed by the P2 subunits of two neighbouring coat proteins) while the other is a trimer involving the S and P1 subunits of three neighbouring coat proteins. (TIF) [file pcbi.1003331.s001.tif]

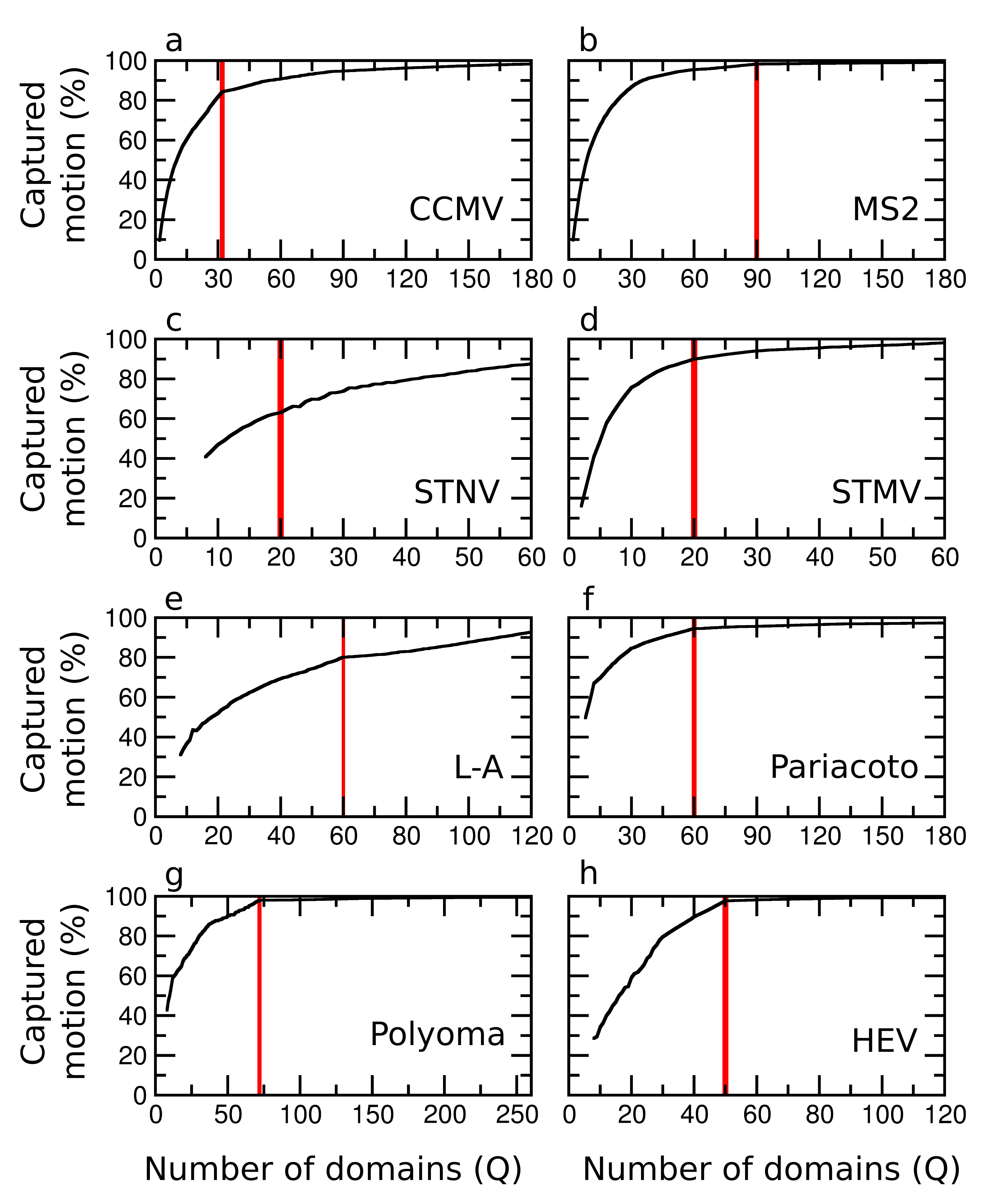

Supplement: Figure S2 — Fraction of overall capsid motion (mean square structural fluctuations) that can be ascribed to the pure rigid-like movements of the quasi-rigid domains. For each value of we considered the domain subdivision which minimizes the geometric strain. Panels a-h refer respectively to: CCMV, MS2, STNV, STMV, L-A virus, Pariacoto virus, polyoma virus and HEV. (TIF) [file pcbi.1003331.s002.tif]

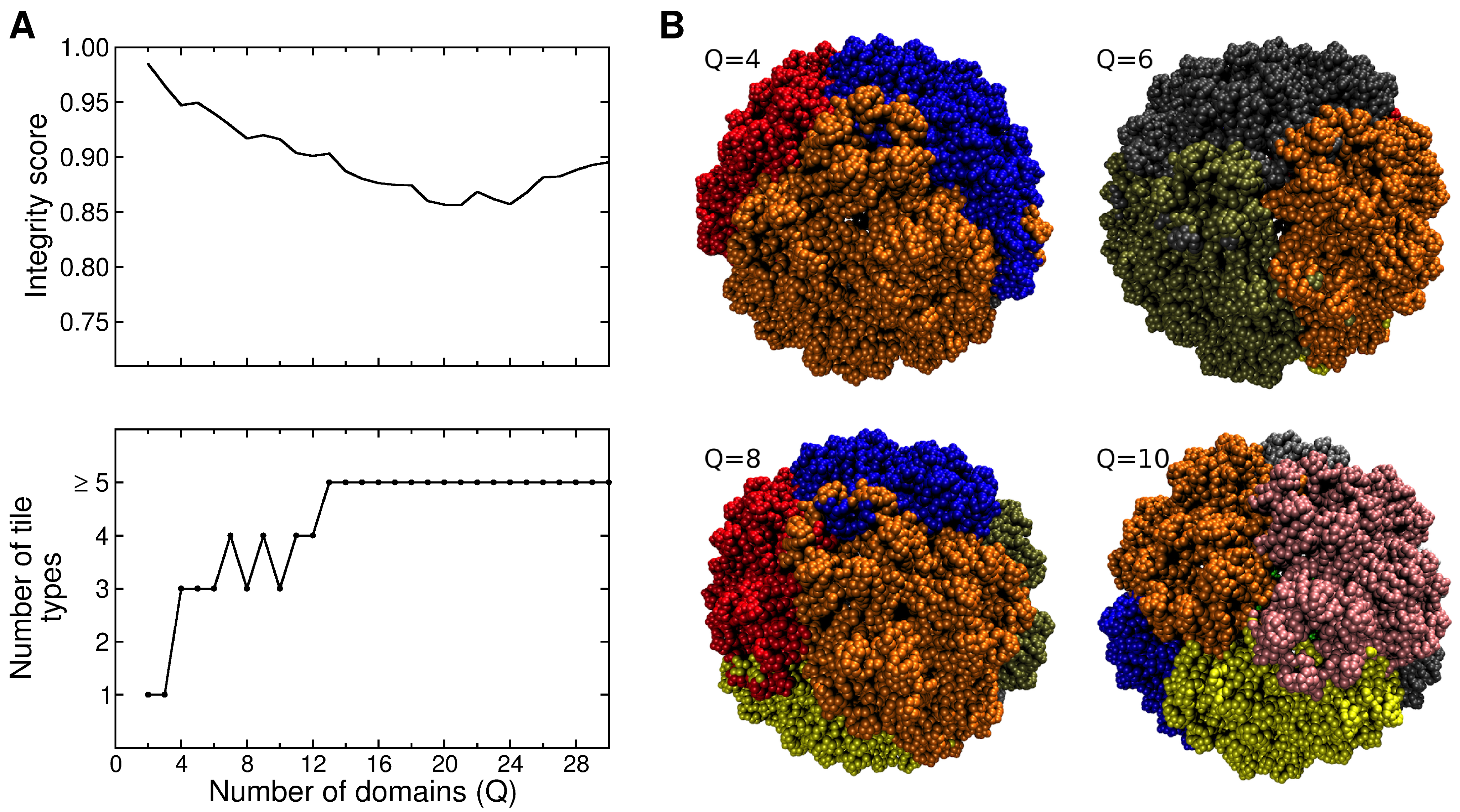

Supplement: Figure S3 — Suboptimal decompositions of CCMV. Panel A shows a close-up of the CCMV profiles for the integrity score and number of tile types for subdivisions from up to 30 quasi-rigid domains. Panel B illustrates non-optimal quasi-rigid decompositions of CCMV. The subdivisions correspond to partitions into very few domains as indicated by the label. For each of these subdivisions the number of different tile type is large and ranges from 3 to 4. For simplicity we therefore used a different color for each domain rather than a different color for domain type as in the figures in the main text. (TIF) [file pcbi.1003331.s003.tif]

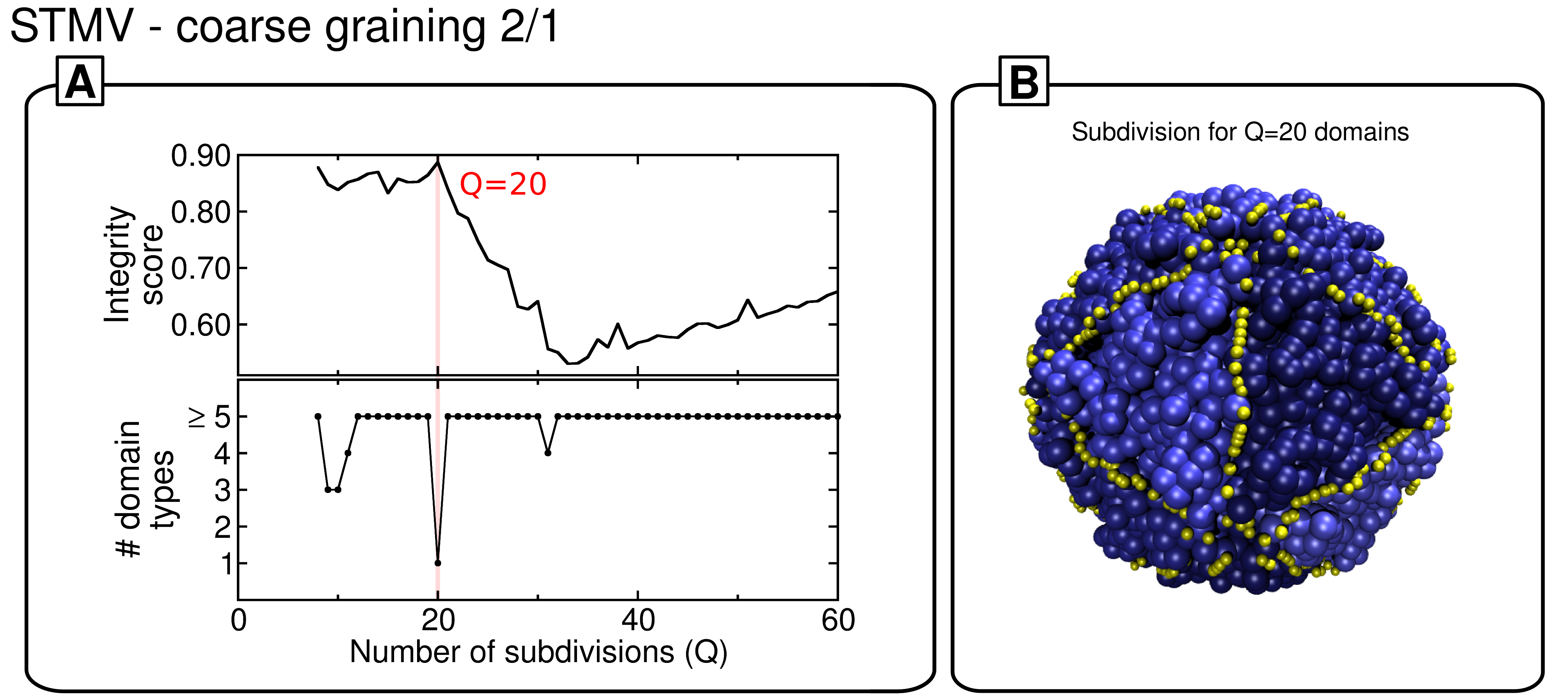

Supplement: Figure S4 — Structural coarse-graining and robustness of quasi-rigid domain decompositions. Optimal subdivision of the STMV capsid into 20 quasi-rigid domains obtained by using the coarse-grained ENM where only every other atom is retained, see Methods. The profiles of various order parameters for the subdivison are shown in box A. The resulting coarse-grained subdivision is shown in box B and is practically indistinguishable from the one given in Fig. 3 where all atoms were retained. (TIF) [file pcbi.1003331.s004.tif]

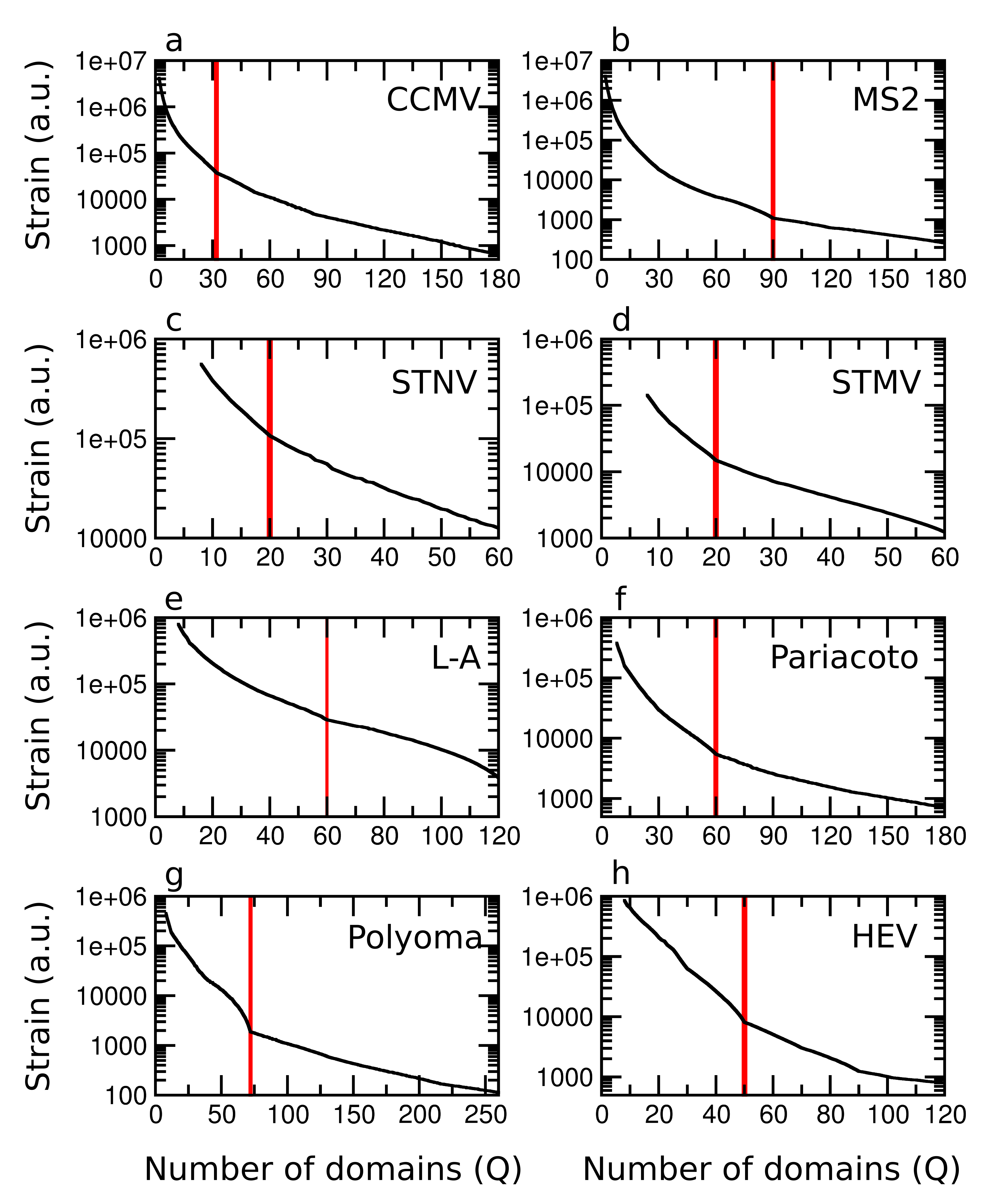

Supplement: Figure S5 — -dependence of the miminized geometric strain. Panels a-h refer respectively to: CCMV, MS2, STNV, STMV, L-A virus, Pariacoto virus, polyoma virus and HEV. Notice that at the value of corresponding to the optimal subdivision (highlighted by the red band) there is usually a kink. The latter signals the change of the slope of the strain curves when the “innate” number of subdivisions is crossed. (TIF) [file pcbi.1003331.s005.tif]
